# Internal fit and marginal adaptation of all-ceramic implant-supported hybrid abutment crowns with custom-milled screw-channels on Titanium-base: in-vitro study

**DOI:** 10.1186/s12903-024-04810-9

**Published:** 2024-09-12

**Authors:** Marwa Wagih Zaky Fouad Fakhr, Hesham Alansary, Eman Ezzat Youssef Hassanien

**Affiliations:** Fixed Prosthodontics Department, Faculty of Dentistry, Cairo University, 11 EL-Saraya St. Manial, Cairo, 11553 Egypt

**Keywords:** CAD/CAM, Lithium disilicate, Replica, Stereomicroscope, Ultra translucent zirconia

## Abstract

**Background:**

Advancements in digital dentistry helped in custom-milling screw-channels in implant-supported restorations; however, the fit of these restorations is still unclear especially for contemporary computer aided designing/computer aided manufacturing (CAD/CAM) materials. This study aimed to compare the internal and marginal fit of Ultra translucent multilayered zirconia versus lithium disilicate implant-supported hybrid abutment crowns (HACs) constructed with custom-milled screw-channels on Titanium-base.

**Materials and methods:**

A total of 24 HACs with custom-milled screw-channels were constructed from lithium disilicate (Group LDS) and Ultra translucent multilayered zirconia (Group UT) using digital workflow (*n* = 12). The internal and marginal gaps of HACs on their corresponding Titanium-bases were assessed using replica technique and stereomicroscope, respectively. After testing for normality, quantitative data were expressed as mean and standard deviation and compared using independent t-test at a level of significance (*P* ≤ 0.05).

**Results:**

There was no statistically significant difference between Group LDS and Group UT in terms of marginal and internal fit. The internal and marginal gaps in both groups were within the accepted values reported in literature.

**Conclusions:**

UT and LDS HACs with custom-milled screw-channels demonstrated comparable and acceptable internal fit and marginal adaptations to Ti-base, which lied within the range reported in literature.

## Background

Implant dentistry has made significant advancements in recent years allowing it to be a reliable option for restoring missing teeth. However, for implant-supported restorations to be successful, it is essential to select the appropriate superstructure [1]. Non-segmented hybrid abutment crown (HAC) is a new approach that involves bonding a one-piece crown with a screw-channel (screw access hole) to a titanium-base (Ti-base) and then screwing them all to the implant fixture [2]. The screw-channel serves as a vent hole for facilitating the screw entry and creating an escape route for excess cement [3]. The term "screwmentable" has been used to describe these combination prostheses [3].

The fabrication process of HAC using digital workflow involves scanning to capture the implant emergence profile, followed by employing a CAD software program to design the virtual crown in addition to designing the screw-channel at the desired position, size and angulation, followed by restoration manufacturing [3]. The HAC restoration is then cemented extra-orally to Ti-base, and the whole assembly is then screwed to the implant fixture intra-orally [3].

This hybrid retention mechanism (cemented and screwed) granted them retrievability and better soft and hard tissue stability, where extra-oral cementation allowed better security at the implant-abutment interface, improved light curing of the restoration margins before screwing, and eliminated the risk of any subgingival residual cement [4, 5] compared to cement-retained implant-supported restorations. HAC also had the ability to customize the emergence profile and enhance the esthetic outcome [4, 5].

Compared to screw-retained restorations, HACs can improve stress distribution due to the presence of the cement layer, which may reduce the strain on the prosthetic components, minimize stress on supporting implants, and ensure the passive fit of the suprastructure on the Ti-base [6].

The Ti-base used with HAC had the advantage of enduring significant occlusal forces due to its high bending moments, which can enhance the fracture resistance of ceramic superstructure and overcome its brittle nature [7]. It can also help protect the metallic implant surface and the material integrity, as titanium-to-titanium contact at the implant-abutment interface produces exceptional stability [8]. Ti-base was also found to overcome some limitations of the prefabricated titanium abutments, such as their standard diameter and average finish line position, which might not necessarily suit the natural contour of the surrounding tissues [1, 5, 9] and adversely affect the peri-implant soft tissues [10] with high risk of metal display [4, 11]. Furthermore, it can create a strong connection between the implant and HAC without eroding the implant surface or causing neck fractures, which are common complications associated with zirconia abutments [7].

Implant superstructures can be fabricated from variety of materials; such as lithium disilicate, which offers high esthetics, marginal accuracy, good internal fit, high fracture load, biocompatibility and excellent bondability with high clinical success in posterior implant-supported restorations [8, 12, 13].

Recently, Ultra translucent multilayered zirconia was used for implant superstructure construction especially in the esthetic zone. It combined high mechanical properties and biocompatibility, with high esthetics [14]. It also offered high flexural strength and fracture toughness, whereas its translucency and multilayered feature helped mimic the shade gradient characteristics of natural teeth [14–16].

For HAC construction using CAD/CAM technology, restorative materials are usually offered in the form of blocks with prefabricated screw-channels to fit the Ti-base, known as meso-blocks. However, these blocks are not commercially available for all dental materials, which limited the use of restorative materials in the construction of HACs. In addition, their use will necessitate extra storage space for these blocks. Furthermore, when considering the cost and practicality of utilizing blocks with prefabricated screw-channels versus regular blocks or blanks without screw-channels, it becomes evident that the latter option is more cost-effective. Moreover, in a study conducted by Kordi AWM, et al. [17], they found that HACs made from lithium disilicate blocks with prefabricated screw-channels and placed on Ti-bases did not ensure greater fracture resistance or better marginal adaptation when compared to those made from solid blocks and placed on conventional Ti- abutments.

The advancement of CAD/CAM systems and the introduction of 5-axis milling machines, allowed milling the whole restoration with a customized screw-channel with high accuracy. This advantage helped overcome the previously mentioned limitations, and widened the spectrum of CAD/CAM restorative materials that can be used [2, 18] for HACs construction to include those supplied as blanks or blocks with no prefabricated screw-channels, pressable materials that can be pressed from milled HACs wax-patterns [19] and 3D printed materials [20].

Additional advantages of HACs with custom-made screw-channels is that they grant the operator control on the position, diameter and angulation of the screw-channel [6], which is not possible with blocks with prefabricated screw-channels. Customizing the screw-channel position and angulation can help enhance the esthetics by moving the channel opening to less apparent positions [21].

Clinical success of implant-supported restorations does not depend only on the materials used; the appropriate internal and marginal fit of the restoration is also an important factor. Discrepancies in the internal fit between Ti-base and HAC may affect the cement flow and subsequently the restoration retention [22]. It might also increase the cement thickness, with increased stress concentration in this weak link, which could negatively affect the mechanical stability of the prosthesis, particularly zirconia that have relatively low bondability [23]. Additionally, proper internal adaptation can minimize the risk of micro-cracks in all-ceramic restorations by reducing the polymerization shrinkage of the cement layer [24, 25]. However, marginal discrepancies between HACs and Ti-bases will result in a gap, which can act as a bacterial reservoir that stimulates and accelerates plaque formation, leading to inflammatory reactions in the peri-implant tissues [7, 26].

Replica technique has been commonly used to test restorations’ internal fit, offering a non-destructive, simple, less expensive, easy, and reliable method with acceptable accuracy, which can be used both in-vitro and in-vivo [25, 27, 28]. The direct view technique of the external marginal adaptation using stereomicroscopes, digital microscopes and scanning electron microscopes was also used in research because it was found reliable, fast, easy, and does not involve restoration destruction [29, 30].

Several studies have tested the fit between the Ti-base and the implant fixture. However, data addressing the fit between HAC, whose screw-channels are custom-milled using 5-axis milling machines and Ti-base are scarce. Although HACs made from monolithic zirconia and lithium disilicate showed high clinical success rates at follow up periods reaching up to 3 years with reduced chipping and fractures [8], studies comparing their marginal and internal fit were deficient. Thus, the aim of the present study was to compare Ultra translucent multilayered zirconia HAC and lithium disilicate HAC fabricated with custom-milled screw-channels in terms of internal fit and marginal adaptation to Ti-base. The null hypothesis was that there would be no statistically significant difference in the internal fit and marginal adaptation of Ultra translucent zirconia and lithium disilicate HACs to Ti-base.

## Materials and methods

### Sample size calculation

Sample size calculation was performed to ensure adequate power to detect statistical significance among the tested groups. Calculations were based on the data obtained from the study conducted by Alves KAF, et al. [18] with 0.05 alpha level of significance and power of 80% using a statistical software (PS: Power and Sample Size Calculation, Vanderbilt University), rendering 12 specimens per group.

### Specimens’ preparation 

Twelve medium-sized titanium implant analogues (Dentaurum Implants GMBH, Ispringen, Germany) with internal hex were used in the present study. Each analogue was vertically embedded within an epoxy resin base (Kemapoxy 150, CMB, Egypt) to facilitate their handling and digital scanning. The analogue’s upper edge was 2 mm above the epoxy resin base to facilitate the marginal assessment.

The upper edge of each epoxy resin base was marked by two indentations prepared using a cylindrical round-ended diamond rotary cutting instrument (No. 880.305S Intensiv, Viganello-Lugano, Switzerland), at the buccal and mesial sides of the epoxy resin base to act as landmarks to facilitate scanning and prevent tracking loss, which might occur due to the smooth, circular and relatively transparent surface of the resin base [31].

Digital scanning was performed using TRIOS 4 intraoral scanner (3Shape, Copenhagen, Denmark) aided with a scan body (Dentaurum Implants GMBH, Ispringen, Germany) which was screwed to the implant analogue using hand torque following the manufacturer’s instructions [31].

**Fig. 1:**
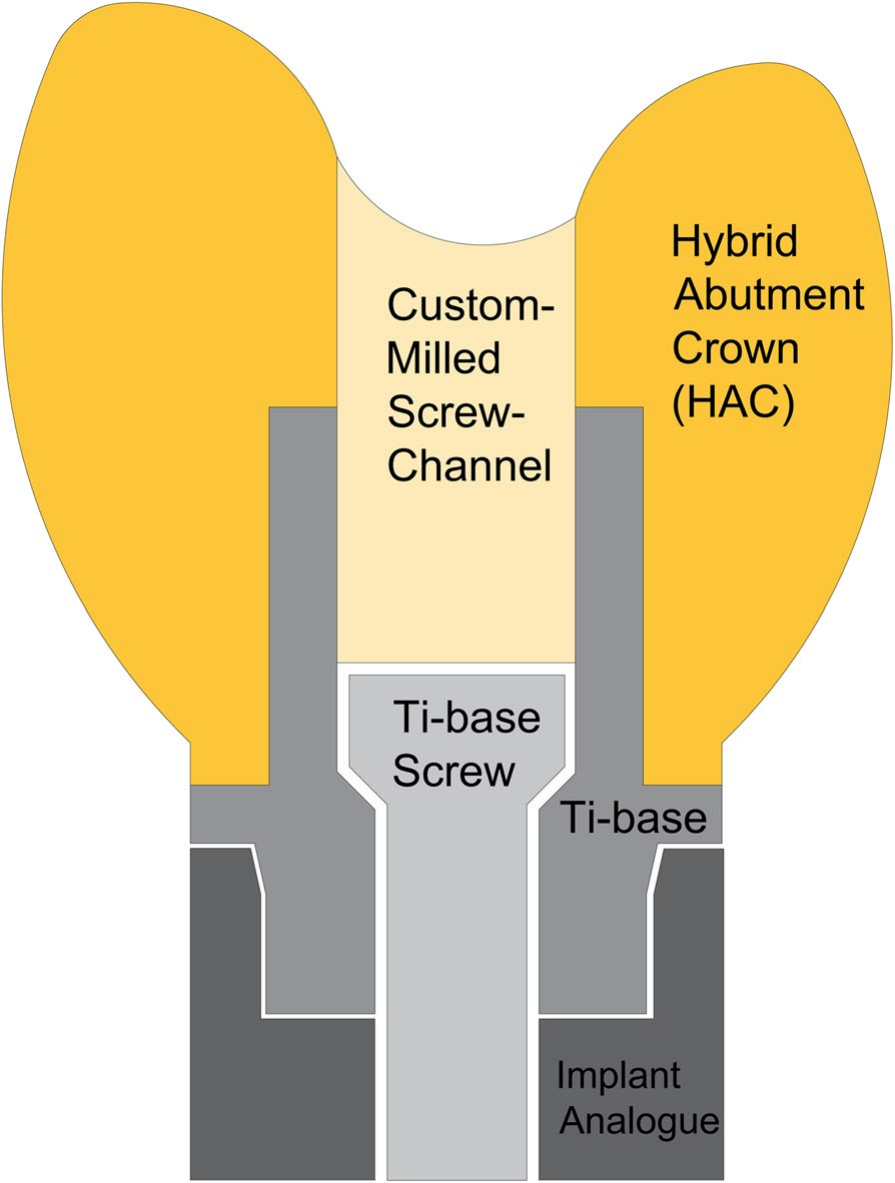
Schematic representation of the HAC seated on Ti-base that is screwed to the implant analogue

**Fig. 2:**
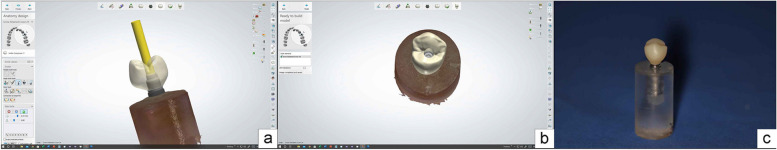
Designing HAC (**a**: The position and orientation of the screw-channel, **b**: The completed design) and the final HAC seated on the Ti-base (**c**)

Twelve scans were completed to fulfil the calculated sample size and exported as Standard Tessellation Language (STL) files to a CAD (Computer-Aided Designing) software (3Shape, Copenhagen, Denmark), where each scan received maxillary left first premolar HAC design (Fig. 1). The Ti-base was selected from the CAD software library according to the implant type [6]. All designs had the same parameters comprising a 50 µm cement gap [32, 33] and a straight screw-channel emerging from the middle of the occlusal surface. A single skilled operator performed scanning and designing to ensure standardization and minimize any discrepancies [34] (Fig. 2a,b).

Each completed design was then exported as an STL file to CAM (Computer-Aided Manufacturing) software (MillbBox CAM software, CIMsystem, Italy) for nesting. Each design was used to mill one HAC from IPS e.max CAD block having shade A2 (LT A2 C 14; Ivoclar Vivadent, Schaan, Liechtenstein) and one HAC from Ultra translucent multilayered zirconia disc having shade A2 (A2 T14 Collar; Katana, Kuraray Noritake, Tokyo, Japan,) using a high-precision 5-axis milling machine (ED5Xz, EMAR, Egypt); resulting in 12 HACs for each group (*n* = 12).

IPS e.max CAD HACs (Group LDS) were wet milled following the manufacturer’s recommendations, whereas Ultra translucent multilayered zirconia HACs (Group UT) were dry-milled and 25% larger than the desired final size of the specimen according to the manufacturer’s instructions to compensate for the shrinkage that would occur during sintering [7].

After milling, both UT zirconia blank and LDS blocks were removed from the milling machine and the connector areas (sprues) were separated from the restorations using diamond discs (Brasselar, USA) [19]. The sprue attachment area was then finished and polished to render a smooth surface, while avoiding contacting the margins of the restorations to avoid any accidental damage or marginal over-thinning that might affect the outcomes tested.

All restorations in both groups were then checked carefully using a magnifying lens, where they were all found free of defects, cracks and margin chippings. All restorations were then cleaned in an ultrasonic cleaner (CODYSON, CD-4820, China) and air-dried to eliminate any residues [23].

Following the manufacturers’ instructions, LDS restorations were crystallized (Programat EP3010 furnace, Ivoclar Vivadent, Schaan, Liechtenstein), whereas UT restorations were sintered (Wiessen Zirconia sintering furnace, Germany).

Finally, each Ti-base was screwed to its corresponding analogue and each HAC restoration was checked individually on its corresponding Ti-base for fit, seating, and marginal adaptation verification, where they all showed proper adaptation and none of them was discarded from the study. (Fig. 2c).

### Assessment of internal fit using replica technique

The Ti-base screw-channel was sealed using polytetrafluoroethylene tape to avoid the impression material impingement in the channel and to confine the light-body impression material film to the cement gap between the Ti-base and HAC. On the contrary, the HAC screw-channel was not sealed to help the excess material escape and to avoid the backpressure that might be generated during seating [33]. The Ti-base was lubricated using a thin layer of lubricant material (Vaseline) to facilitate the removal of the superstructure holding the replica without distorting.

A mix of fast-set polyvinyl siloxane light-body impression material (Elite HD +, Zhermack Dental Inc., Italy) was injected inside each HAC [35], which was then seated on its corresponding Ti-base and fixed in position using steady finger pressure for five minutes until the silicone impression material was fully polymerized [26, 32].

After complete setting, the HAC was removed, and the light silicone film was checked for continuity and lack of distortion. Afterwards, a more viscous polyvinyl medium-body silicone impression material (Panasil soft, Kettenbach, Escheburg, Germany) with a different color was mixed and injected into the fitting surface of HAC on top of the light-body silicone film to stabilize it [29, 30] and facilitate its separation from HAC after setting [18].

**Fig. 3:**
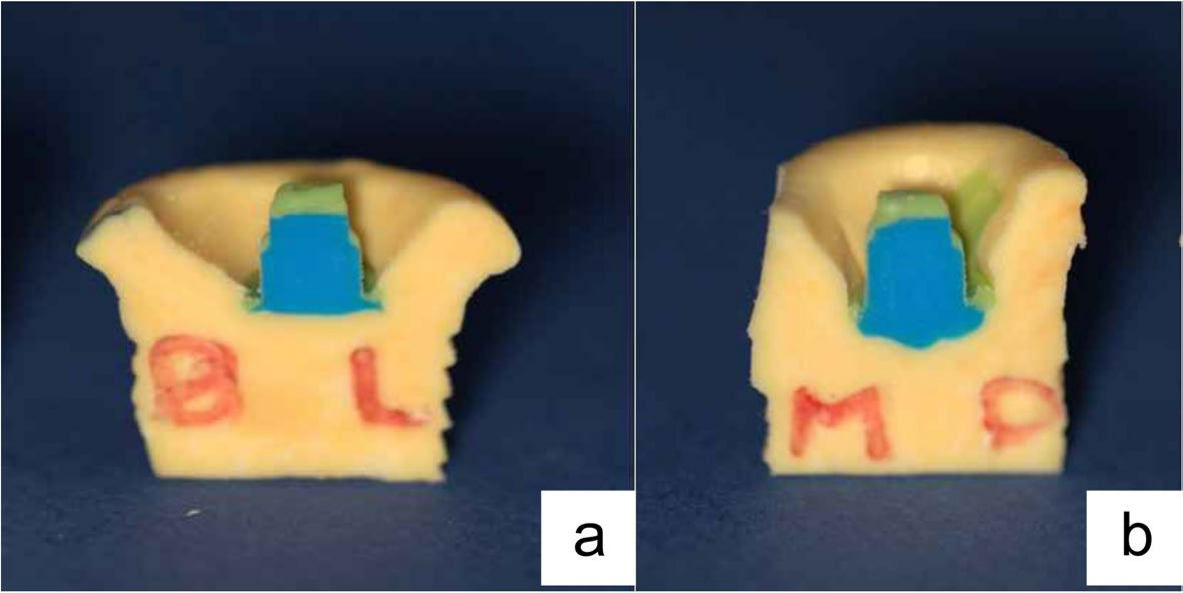
Polyvinyl silicon replica (**a**: The first replica sectioned in a bucco-palatal direction to analyze the buccal and lingual surfaces, **b**: The second replica sectioned in mesio-distal direction to analyze the mesial and distal surfaces, the blue color: medium body polyvinyl siloxane material, the green color: the film of light body polyvinyl siloxane material)

Two replicas were made for each HAC and sectioned using a sharp scalpel (blade #15, Wuxi Xinda Medical Device Co Ltd., China) in a bucco-palatal and mesio-distal direction respectively [26] (Fig. 3). The blade was replaced after each sectioning [18].

**Fig. 4:**
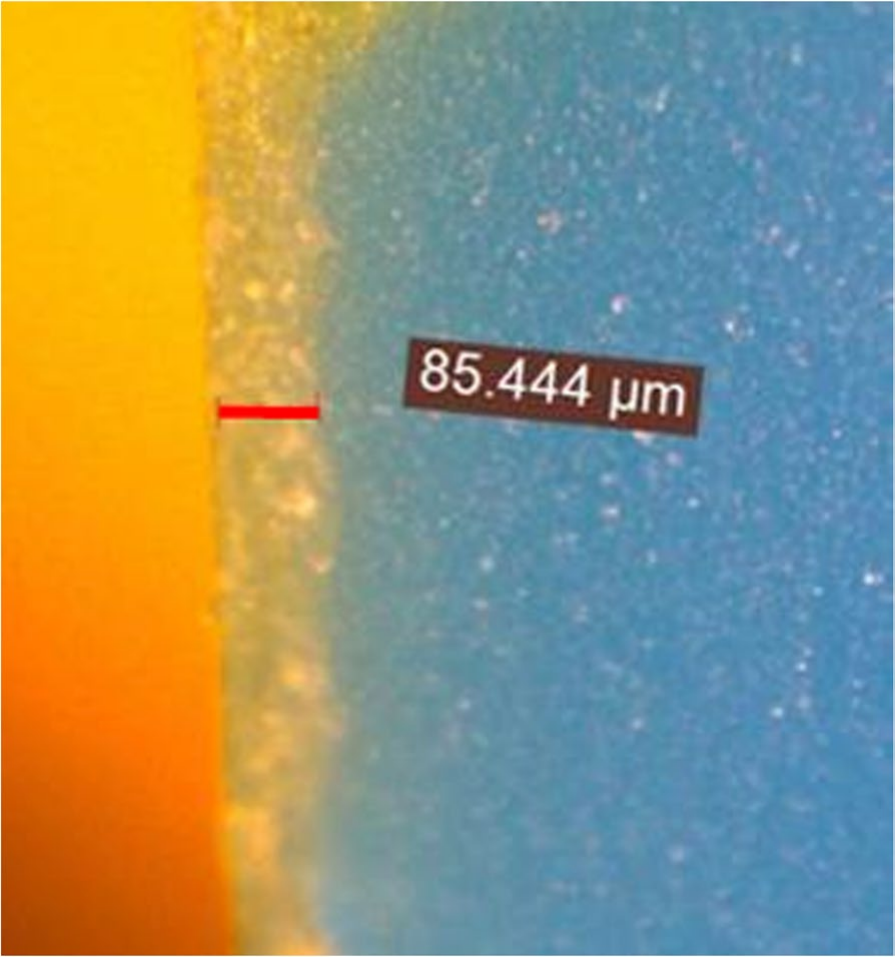
Stereomicroscopic image (50 × magnification) for measuring the internal gap on silicone replica film:(The blue color: medium body polyvinyl siloxane material, the light green color: film of light body polyvinyl siloxane material, The red line denotes the internal gap)

Each sectioned replica was analyzed using a stereomicroscope (Leica DFC290, Leica Microsystems Ltd., Germany) at 50 × magnification, where 3 equidistant measurement points were recorded (P1: cervical, P2: middle, and P3: occlusal) for each surface (buccal (B), palatal (P), mesial (M), and distal (D), to achieve a consistent, reliable and reasonable estimation of the gap size [17, 18, 33, 35] (Fig. 4).

### Assessment of marginal adaptation

**Fig. 5:**
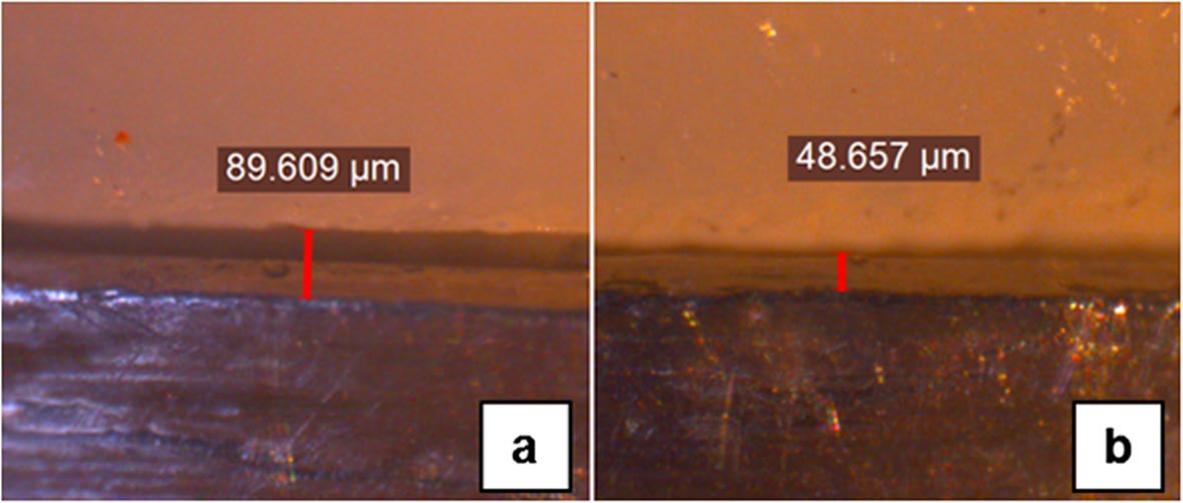
Stereomicroscope images (50 × magnification) for measuring the marginal gap distance (**a**: in Group LDS, **b**: in Group UT)

The external vertical marginal gap between each HAC and its corresponding Ti-base was measured in both tested groups without cementation [36], using the same stereomicroscope at 50 × magnification. One calibrated investigator, who was blinded to the experimental groups, measured the marginal gap [23] at four positions: mid-buccal, mid-palatal, mid-mesial, and mid-distal [17, 26, 37, 38] (Fig. 5). In order to standardize the measurement points for all specimens, a consistent location in the middle of all four surfaces (buccal, palatal, mesial and distal surfaces) of the epoxy resin base was marked using a permanent indelible marker, and the marginal gap was assessed at a points corresponding to those marked locations [39].

Each specimen was stabilized using a special holding device to maintain the superstructure on the Ti-base while measuring the marginal gaps [19, 35]. All the measurements were meticulously repeated three times, and average values were taken [23, 35].

## Statistical analysis

An expert statistician, who was blinded to the tested variables, analyzed the quantitative data collected using SPSS software (v.20, IBM, USA). All data were explored for normality using Shapiro Wilk and Kolmogorov–Smirnov normality test, which revealed parametric data distribution. Data were presented as mean and standard deviation (SD) and compared using independent t-test. *P*-value ≤ 0.05 was considered statistically significant.

## Results

The mean internal and marginal gap values and their standard deviation (SD) were reported in Table 1. No statistically significant difference was found in the mean internal and marginal gaps between the tested groups. Group LDS showed lower internal gap values than Group UT, While Group UT showed lower marginal discrepancy values than Group LDS.

**Table 1:** Results of the internal and marginal gaps

**Internal gap values (µm)**								
**Group**	**N**	**Min**	**Max**	**M**	**SD**	**MD ± SEM**	**95% CI**	***P***-**value**
**LDS**	12	31.25	58.50	43.89	8.02	27.73 ± 4.30	18.8—36.6	0.15
**UT**	12	53.75	94.00	71.62	12.56			
**Marginal gap values (µm)**								
**Group**	**N**	**Min**	**Max**	**M**	**SD**	**MD ± SEM**	**95% CI**	***P***-**value**
**LDS**	12	81.00	100.00	94.00	6.51	50.23 ± 2.55	44.9—55.5	0.79
**UT**	12	33.50	52.40	43.77	6.00			

*N* number, *Min* minimum, *Max* maximum, *M* mean, *SD* standard deviation, *MD* mean difference, *SEM* standard error mean, *CI* confidence interval, *P*-Value probability level is significant at *P* ≤ 0.05

## Discussion

The null hypothesis of the present study was accepted, where there was no statistically significant difference among the tested groups regarding the internal and marginal gaps.

Dental implants became a crucial part of prosthodontics. However, evidence regarding the clinical reliability of HACs with custom-milled screw-channels in the posterior region, in terms of internal and marginal fit to the underlying Ti-base is lacking. Therefore, the present study tested HACs made from esthetic ceramics (LDS compared to UT) to help clinicians choose the material that can offer the best adaptation [40]. The aim of the present study was to test the effect of these two different materials on the marginal and internal adaptation of HACs. Hence, similar manufacturing technique (involving customized screw-channels) was employed for both materials to limit any confounding factors that might affect the results such as using blocks with prefabricated screw-channels.

In the present study, superstructures restoring maxillary 1st premolars were the restorations tested, due to their high esthetic importance and frequent loss of these teeth [2, 41]. Both tested materials used for HACs construction had A2 Shade to conform to one of the most prevalent shades among population. [42]. In the present study, full digital workflow was carried out for its simplicity, time-saving benefits, accurate fit and reduced need for manual interventions compared to conventional workflow [5, 8, 12, 19, 35, 43–45]. Intraoral scanner was implemented because of its proven effectiveness and accuracy [46], with the scan body aiding in detecting the spatial position of the analogues as employed in clinical cases [44]. Scanning the scan body instead of directly scanning the Ti-bases helped to avoid the errors resulting from the metal reflection or powder scanning, which could affect the restoration fit [47].

In the present study, HAC was designed fully contoured instead of a simple framework to simulate the clinical situation to allow more realistic results and to decrease the risk of chipping that may occur to coping’s thin margins [7]. The cement gap thickness was set to 50 μm, in accordance with other studies such as Ferrairo BM, et al.; Pacheco ND, et al. [32, 33], to offer the best marginal fit. Designing of the screw-channels was adopted because this advantage enabled the use of Ultra translucent multilayered zirconia blanks, where, as to date, there are no available meso-blocks for this material [2, 18]. HACs with custom-made screw-channels were found to be more effective than cement-retained restorations in reducing the vertical marginal discrepancies as documented by Yildiz et al. [3]. Although creating a screw access channel in lithium disilicate crowns could potentially reduce their axial load capacity compared to those without such channels, the studies have found that the size of the screw access channel does not have a significant impact on the load-bearing capacity of the crown [48].

The advancements in CAD technology made the designing of the custom-milled screw-channels feasible and accurate. Many CAD software; such as Exocad DentalCAD (Exocad GmbH) [2, 20], Ceramill (Amann Girrbach) [21], Cerec Omnicam (Dentsply Sirona) [8] were used in previous studies to design HACs with custom-milled screw-channels, in addition to, 3Shape CAD software used in the present study.

The scanner and designing software of the same company (3Shape) were used in the current study; to avoid multiple transfer of the data from one software to another, which could cause errors resulting in decreased accuracy [49]. A 5-axis milling machine was utilized, to ensure high milling accuracy and accessibility, to help precisely mill the screw-channels in both groups [2]. The design of UT superstructures was increased by 25% to compensate for the shrinkage during the sintering process. However, the LDS design was not enlarged as the milling software considers the densification factor (0.2%—0.3%) that occurs during the crystallization process [18].

A stereomicroscope was used in this study to test both the internal fit and marginal adaptation of the restorations because it provides a high degree of accuracy in gap detection, in addition to being easy to use and not costly [50]. Measuring the gap of the specimens while they were uncemented was performed to eliminate the variables that can influence the results such as the cement type and film thickness [36, 51].

Regarding the internal fit, previous studies recommended an internal gap not more than 50 to 100 µm for indirect restorations cemented by conventional cements and an internal gap not more than 200 to 300 µm for indirect restorations cemented by adhesive cement. Recently, it has been recommended that the internal gap for adhesive cement should not exceed 100 µm [32]. Other studies suggested that a 150-µm gap can be considered clinically acceptable misfit value for implant-supported restorations [7].

In our study, there was no statistically significant difference between the tested groups in terms of internal fit, with gap values being within the clinically accepted range (150 µm) [7]. This might be due to the standardization that was employed throughout the study for both groups. In addition, using a virtual cement thickness of 50 µm might also contribute to this result, as it was proved effective in providing the minimum adequate space for cement accommodation [52]. Although there was no statistically significant difference, UT showed a slightly higher mean internal gap than LDS. This minor difference could be attributed to the different nature of the materials tested and the difference in the milling tools typically used for either material [40].

These results were in accordance with those of Demirel et al. [20], who examined the internal fit of HACs with custom-milled screw-channels made of 3D printed composite resin (Crowntec), 3D printed hybrid composite resin (VarseoSmile Crown Plus), reinforced composite resin blocks (Brilliant Crios) and polymer-infiltrated ceramic network blocks (Vita Enamic) on Ti-bases surfaces using triple scan technique. Their findings were comparable to our results being within the clinically accepted range reported in literature.

On the other hand, the results of the present study disagreed with Güngör et al. [54], who found significantly higher internal gap values in monolithic zirconia (InCoris TZI) crowns than in lithium disilicate (IPS e.max CAD) crowns using replica technique. This disagreement might be attributed to the difference in the scanning device, designing software and milling machine compared to our study. It might also be due to the difference in the restored tooth and the cement thickness utilized, where they used 80 µm for mandibular right first molar restorations.

The present results also disagreed with Elbanna and Alqutub [53], who found that zirconia (Ceramill Zolid HT) crowns showed significantly better internal fit on zirconia abutments than lithium disilicate (e.max CAD) crowns, when using replica technique. This difference might be due to the difference in the CAD software used (Exocad) with a 30 µm cement gap thickness and the used abutment which was custom-milled.

To date, there has been an ongoing debate regarding the determination of the maximum clinically acceptable marginal gap for both tooth- and implant-supported restorations. The reported values in the literature span from 50 to 200 μm, as documented by various researchers [7, 19, 51]. According to the American Dental Association, the clinically acceptable fit for an indirect restoration falls within the range of 50 to 100 µm [55]. However, a study by McLean JW & von Fraunhofer JA [56] based on over a thousand restorations concluded that a marginal gap of less than 120 μm is clinically accepted.

In the present study, there was no statistically significant difference between the groups regarding marginal adaptation, while being within the acceptable clinical values (120µm) [5, 35]. This might also be due to the appropriate standardization of the scanning and designing parameters in both groups. The minor difference between LDS and UT might be due to the variation in their structural composition causing lower edge stability in lithium disilicate than in zirconia restorations, with higher marginal discrepancy [57]. In addition to the difference in the milling environment, where UT was dry milled, while, LDS was wet milled, might have affected the marginal accuracy [40].

Our results were supported by Zarone F, et al. [52], who concluded in their review that the marginal accuracy of zirconia restorations was better than its internal fit, which was believed to be due to the shape and size of the CAD/CAM milling burs. Our results also agreed with Riccitiello F, et al. [23], who found no statistically significant difference in the marginal discrepancies between zirconia (Katana Zirconia) and lithium disilicate (IPS e.max CAD) single crowns, with zirconia showing better results.

Our results were in agreement to those of Yildiz P, et al. [3], who found that marginal discrepancy between monolithic zirconia (Upcera-ST, Upcera) abutments with custom-milled screw-channel to Ti-base falls within the clinically accepted range reported in the literature. This also agreed with the results of Kordi et al. [17], who found that the marginal gap value of HACs and crowns made of (IPS e.max CAD) were within the clinically accepted ranges documented in the literature.

However, our results disagreed with Nejatidanesh F, et al. [35], who tested the internal and marginal fit of milled lithium disilicate (IPS e.max CAD) and veneered zirconia (Cercon) restorations on Titanium prefabricated abutments and found that the internal gap of lithium disilicate was significantly larger than that of zirconia, whereas marginal gaps were higher in zirconia than lithium disilicate with no statistically significant difference. This disagreement can be attributed to the difference in the milling machine (Cerec AC), restorative material (Cercon zirconia copings), scanning technique (powder scan), using different cement gap thickness (10µm), and applying a veneer layer.

The limitations of the present study included being in-vitro, which might not fully represent the clinical conditions. Additionally, the measurements were taken before cementation, so any potential increase in marginal and internal gaps after cementation was not evaluated. Another limitation of this study was the lack of testing a control group that employed HACs with prefabricated screw-channel. Hence, it is recommended to conduct further studies to compare the effect of prefabricated and custom-made screw-channels on the marginal and internal fit of HACs. It is also recommended to test the effect of different materials, aging and cyclic loading on the marginal and internal fit of HACs to Ti-bases.

## Conclusions

### Within the limitations of the present study, the following can be concluded

UT and LDS HACs with custom-milled screw-channels demonstrated comparable and acceptable internal fit and marginal adaptations to Ti-base, which lied within the range reported in the literature.

### Clinical relevance

When restoring missing maxillary premolars with implant-supported restoration, using a monolithic hybrid abutment crown with a custom-milled screw-channel made of Ultra translucent multilayered zirconia or lithium disilicate can be considered viable options in terms of internal fit and marginal adaptation to Titanium base. However, it is crucial to consider that the use of this design should be supported by clinical studies validating its performance.

## Data Availability

The data that support the findings of this study are available from the corresponding author upon reasonable request.

## Abbreviations

CAD/CAM: Computer aided design/ computer aided manufacturing
HACs: Hybrid abutment crowns
Group LDS: Lithium disilicate
Group UT: Ultra-translucent multilayered zirconia
Ti-base: Titanium base
PS: Power and Sample Size
STL: Standard Tessellation Language
CAD: Computer aided designing
SD: Standard deviation
N: Number
Min: Minimum
Max: Maximum
M: Mean
MD: Mean difference
SEM: Standard error mean
CI: Confidence interval
*P*-Value: Probability level is significant at *P* ≤ 0.05
